# TLR-4 engagement of dendritic cells confers a BST-2/tetherin-mediated restriction of HIV-1 infection to CD4^+^ T cells across the virological synapse

**DOI:** 10.1186/1742-4690-10-6

**Published:** 2013-01-11

**Authors:** Fabien P Blanchet, Romaine Stalder, Magdalena Czubala, Martin Lehmann, Laura Rio, Bastien Mangeat, Vincent Piguet

**Affiliations:** 1 Department of Dermatology and Wound Healing, Institute of Infection and Immunity, Cardiff University School of Medicine, 3rd Floor, Glamorgan house, Heath Park, Wales, Cardiff, CF14 4XN, United Kingdom; 2 Dept. of Microbiology and Molecular Medicine, Medical School of Geneva, Geneva, Switzerland

**Keywords:** Human immunodeficiency virus-1 (HIV-1), Bone marrow stromal cell antigen-2 (BST-2)/tetherin, Dendritic cells, Lipopolysaccharide (LPS), Interferon-alpha (IFN-α)

## Abstract

**Background:**

Dendritic cells and their subsets, located at mucosal surfaces, are among the first immune cells to encounter disseminating pathogens. The cellular restriction factor BST-2/tetherin (also known as CD317 or HM1.24) potently restricts HIV-1 release by retaining viral particles at the cell surface in many cell types, including primary cells such as macrophages. However, BST-2/tetherin does not efficiently restrict HIV-1 infection in immature dendritic cells.

**Results:**

We now report that BST-2/tetherin expression in myeloid (myDC) and monocyte-derived dendritic cells (DC) can be significantly up-regulated by IFN-α treatment and TLR-4 engagement with LPS. In contrast to HeLa or 293T cells, infectious HIV-1 release in immature DC and IFN-α–matured DC was only modestly affected in the absence of Vpu compared to wild-type viruses. Strikingly, immunofluorescence analysis revealed that BST-2/tetherin was excluded from HIV containing tetraspanin-enriched microdomains (TEMs) in both immature DC and IFN-α–matured DC. In contrast, in LPS-mediated mature DC, BST-2/tetherin exerted a significant restriction in transfer of HIV-1 infection to CD4^+^ T cells. Additionally, LPS, but not IFN-α stimulation of immature DC, leads to a dramatic redistribution of cellular restriction factors to the TEM as well as at the virological synapse between DC and CD4^+^ T cells.

**Conclusions:**

In conclusion, we demonstrate that TLR-4 engagement in immature DC significantly up-regulates the intrinsic antiviral activity of BST-2/tetherin, during cis-infection of CD4^+^ T cells across the DC/T cell virological synapse. Manipulating the function and potency of cellular restriction factors such as BST-2/tetherin to HIV-1 infection, has implications in the design of antiviral therapeutic strategies.

## Background

Dendritic cells (DC) are critical initiators of immune responses after encountering a pathogen at mucosal sites [1]. These cells have potent phagocytic and anti-microbial properties placing them at the interface between innate and adaptive immunity [2]. Pathogens, such as HIV-1, have evolved strategies to counteract DC functions and some DC subtypes, such as myeloid DC (myDC) or Langerhans cells (LC), have been shown to potently restrict HIV-1 infection in the early events of viral entry due to their primary endo/phagocytic functions [3-5]. Several additional obstacles to HIV-1 replication in DC and their subsets have also been identified. Surface receptors, such as langerin [4], cellular restriction factors including SAMHD1 [6,7] and members from the APOBEC3 protein family [8-11] as well as other restriction factors that remain undefined [12,13] can inhibit HIV-1 in the early stages of entry and replication within DC. The stage at which LC (DC subtype) activation occurs is important, as langerin is down-regulated in mature LC [14,15] while APOBEC-3 levels are increased in mature DC [10,16]. This shows that restriction in DC is not static and is highly regulated by the inflammatory environment that can be modulated by the pathogen. In addition, later steps of HIV-1 replication are also compromised in DC, mostly in mature cells ([17-19] and reviewed in [20]). HIV-1 was shown to be able to circumvent some of these restrictions mainly due to accessory viral proteins that were able to antagonize specific cellular restrictive or antiviral activities [21-23]. Among these, viral protein U (Vpu) was recently shown to antagonize the antiviral effect of BST-2/tetherin, an interferon (IFN) inducible protein, able to tether nascent virions to cellular membranes [24,25]. BST-2/tetherin can inhibit a broad range of enveloped viruses [24-30].

Vpu is a critical factor for efficient viral release in several cell types [31,32] (and see an extensive review in [33]). This capacity is dependent on the interaction of Vpu with BST-2/tetherin, leading to its cell surface down-regulation [34-36] and degradation of BST-2/tetherin in some cell types via a β-TrCP-dependent mechanism [37-40]. A very elegant model of the mode of action of BST-2/tetherin was recently proposed, showing that antiviral activity was mainly based on the structural configuration of the protein, rather than the primary sequence, leading to incorporation of parallel homodimers into HIV-1 particles [28]. Furthermore, super-resolution microscopy studies have established that 5 to 6 BST-2/tetherin dimers are sufficient to restrict HIV-1 budding [41]. However, BST-2/tetherin restriction seems to be cell type dependent (to some extent) as DC have been reported as unable to inhibit replication of HIV-1 in the absence of Vpu [42]. Finally, tetherin is also able to counteract cell-to-cell transfer of HIV-1 under some circumstances [43,44].

In our study, we report that both human monocyte-derived dendritic cells (DC) and myeloid DC (myDC) express BST-2/tetherin constitutively, which can be highly up-regulated upon IFN-α treatment, as previously shown in murine DC [45]. We demonstrate that BST-2/tetherin was dramatically down-regulated from the cell surface of infected DC and myDC in a Vpu-dependent manner, but there is no evidence of subsequent protein degradation. However, while IFN-α pre-treatment decreased HIV-1 infection of DC in *cis*, we unexpectedly observed that viral release and infectivity were only slightly affected upon DC infection with *vpu*-deficient HIV-1 (HIV-ΔVpu). Thus, we compared virion localization by confocal imaging of DC infected with HIV-WT and HIV-ΔVpu. While the majority of HIV-WT and HIV-ΔVpu viruses were stained at the DC cell surface, we observed a lack of BST-2/tetherin staining when viruses were localized in intracellular compartments. We identified these compartments as the previously reported surface-accessible tetraspanin-enriched (TEM) compartments [46]. We demonstrate that HIV-WT and HIV-ΔVpu transfer to CD4^+^ T cells, both in *trans* and in *cis*, was not affected even when BST-2/tetherin expression was silenced in DC. Hence, the fact that no restriction could be observed upon DC-mediated viral transfer correlated with the absence of BST-2/tetherin at the virological synapse upon contact with primary CD4^+^ T cells. Finally, by imposing differential levels of restriction activity in DC, we could observe that while IFN-α pre-treatment of DC did not apparently modulate BST-2/tetherin localization and restriction activity, LPS pre-treatment induced a significant re-localization of the restriction factor to the TEM. The observed LPS-mediated BST-2/tetherin re-localization correlated with its polarization at the virological synapse formed between DC and CD4^+^ T cells, with consequent BST-2/tetherin restriction activity being revealed in DC-mediated HIV-1 transfer *in cis* to CD4^+^ T cells.

In conclusion, we have demonstrated that HIV-1 avoids BST-2/tetherin restriction in immature DC, as well as IFN-α treated DC, on the basis of complex trafficking, despite a seemingly conserved role of Vpu in these cells. Strikingly, TLR-4 engagement of DC correlated with a significant up-regulation of BST-2/tetherin-mediated restriction of *vpu*-deficient HIV-1 both in *cis* and in *trans*. Furthermore, we have shown that TEM composition is modulated after LPS-mediated DC maturation with re-localization of BST-2/tetherin to this domain, which could take part in the restricted viral transfer to target T cells initiated from infected mature DC.

## Results and discussion

### HIV-1 Vpu down-regulates cell surface BST-2/tetherin in infected DC

**Figure 1:**
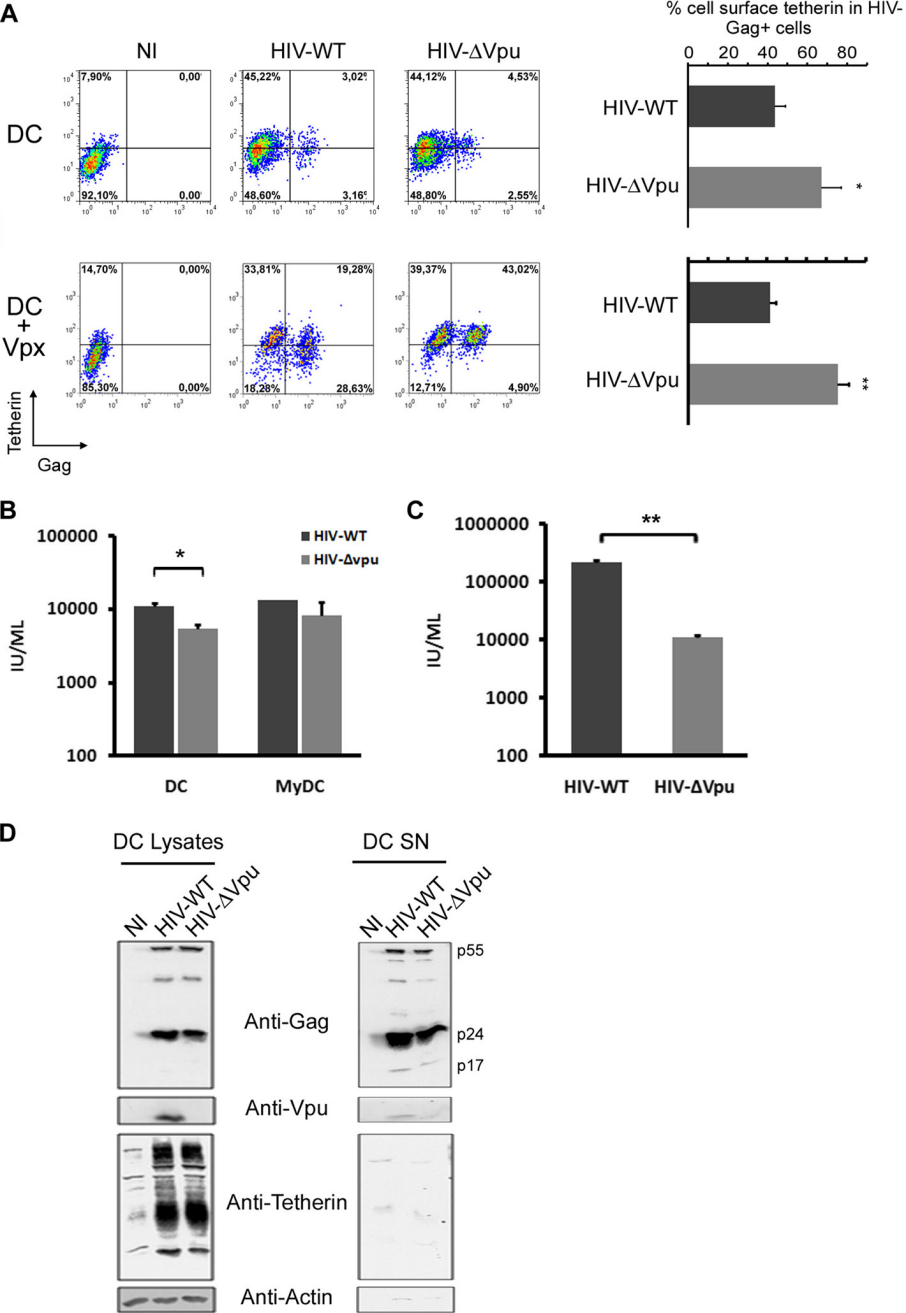
**Vpu-dependent BST-2/tetherin cell surface down-regulation but lack of BST-2/tetherin restriction activity in DC. (A)**. 2 × 10^5^ DC (A, upper panel) and Vpx-transduced DC (A, lower panel) were infected or not (NI) with HIV-WT or HIV-ΔVpu viruses. Infection of DC was scored by FACS analysis of BST-2/tetherin^+^/p24gag^+^ cells, three days post-infection. Percentage of cell surface BST-2/tetherin^+^ in infected DC from 7 independent experiments was calculated (right graph panels). **(B)** TZM-*bl* cells were incubated for 48 hours with 1/10 volume of supernatant from HIV-WT or HIV-ΔVpu infected DC or myDC harvested three days post-infection. Infectious units (IU/ml) were quantified using the β-gal assay obtained from 5 and 3 experiments in DC and myDC respectively. **(C)** Similar experiments (as above) were performed with supernatant obtained from HIV-WT or HIV-ΔVpu infected Hela cells (results from duplicate experiments). Error bars represent the mean ± SD of indicated experiment numbers. **(D)** Lysates and supernatants of uninfected (NI), HIV-WT or HIV-ΔVpu infected DC were subjected to immunoblotting with anti-Gag and anti-BST-2. The presence of Vpu was assessed by immunoblotting with anti-Vpu and anti-actin served as loading control.

BST-2/tetherin cell surface levels were determined by flow cytometry in DC and myDC infected with HIV-1 in the presence or absence of Vpu. Due to a potent post-entry restriction block, DC were particularly refractory *per se* to productive HIV-1 infection even when Vpu was absent (Figure 1A, upper panels), while myDC appeared less refractory to productive infection (Additional file 1: Figure S1A, upper panels). Although, quantification of a significant down-regulation of Vpu-mediated BST-2/tetherin at the cell surface of HIV-infected cells from different donors was possible, the low level of infection renders qualitative measurements more challenging. Therefore, we took benefit from the recently discovered ability of Vpx to circumvent post-entry restriction block in DC [6,7,12] in order to increase levels of HIV-1 infection. Co-transduction of DC with pseudotyped GFP-expressing HIV and Vpx-expressing SIV-derived lentivectors led to robust GFP expression at day 2 and 5 post-infection as shown by FACS analysis (Additional file 1: Figure S2). Given the ability of Vpx to confer susceptibility of DC and myDC to HIV infection, we investigated whether Vpu could down-regulate the level of BST-2/tetherin at the surface of both cell types. DC were left uninfected or infected with X4-tropic full length HIV-1virus (HIV-WT) or a Vpu deleted version (HIV-ΔVpu), concomitantly with the transduction of Vpx-expressing SIV-derived lentivectors. Two days after HIV-WT infection, FACS analysis of HIV-Gag positive cells demonstrated a significant cell surface BST-2/tetherin down-regulation of around 60% in DC (Figure 1A, lower panels) and 70% in myDC (Additional file 1: Figure S1A, lower panels) compared to non-infected (NI) cells. This effect was Vpu-dependent since down-regulation was negligible when cells were infected with HIV-ΔVpu (Figure 1A and Additional file 1: S1A).

BST-2/tetherin was previously shown to greatly reduce viral release and infectivity from cells infected with Vpu-deficient viruses [24,25,47]. To investigate if BST-2/tetherin could also restrict HIV-1 replication in DC subtypes, we analyzed the release of infectious viruses from supernatants of cells infected with either HIV-WT or HIV-ΔVpu viruses. We then analyzed the yield of infectious virions using TZM-bl reporter cells and observed that the absence of Vpu slightly, but significantly, decreased viral infectivity by 2 fold in DC. Although viral infectivity was also lower in myDC infected with HIV-ΔVpu, this decrease was not significant (Figure 1B). Overall, the observed decrease of viral infectivity and viral release from HIV-ΔVpu infected DC was not comparable to the 12 fold decrease of viral infectivity observed in supernatants from HIV-ΔVpu infected HeLa cells (Figure 1C). These data strongly suggest that BST-2/tetherin in DC and myDC was far from being as restrictive as observed in HIV-ΔVpu infected HeLa cells where almost 93% of HIV-ΔVpu release was blocked.

The relative lack of significant BST-2/tetherin restriction activity in DC was confirmed by anti-HIV-Gag immunoblot of cell lysates and supernatants obtained from HIV-WT and HIV-ΔVpu infected cells showing comparable levels of infection (Figure 1D). The same results were obtained with infected myDC (Additional file 1: Figure S1B). Additionally, Vpu-mediated BST-2/tetherin degradation, reported in other cell types [37,38,48], was not demonstrated in lysates of either DC subtypes even with high levels of infection (Figure 1D and Additional file 1: Figure S1B).

### LPS-mediated DC maturation induces BST-2/tetherin clustering and potently inhibits HIV-1 replication

BST-2/tetherin expression, whether constitutive or IFN-induced, has been well-characterized in some cell types [24,25] and even in primary immune cells [49]. However, there is still limited characterization of BST-2/tetherin expression and function in primary and *in vitro* derived myeloid DC (myDC). Thus, we first analyzed surface (non-permeabilized) and total (permeabilized) BST-2/tetherin expression levels in non-treated (NT), IFN-α or LPS treated DC compared to IFN-α-treated 293T cells by FACS. Indeed, 293T cells are an appropriate control for antibody specificity as they are known to lack constitutive BST-2/tetherin expression (Additional file 1: Figure S3A and [24]). As expected, BST-2/tetherin expression levels were increased in 293T cells treated for 24h with IFN-α (Additional file 1: Figure S3A). As shown, DC expressed constitutive levels of BST-2/tetherin that were significantly up-regulated upon IFN-α treatment. LPS appeared to down-regulate BST-2/tetherin cell surface levels while increasing global expression (Additional file 1: Figure S3A). The effect of IFNα- and LPS-mediated up-regulation of BST-2/tetherin in DC and 293T cells was confirmed by Western blotting (Additional file 1: Figure S3B). Of note, a specific smear of BST-2/tetherin at 80-90KDa appeared in IFN-α treated cells but was barely detected in LPS-treated DC (Additional file 1: Figure S3A). This pattern would fit with the presence of highly glycosylated forms of BST-2/tetherin, thus correlating with an increased surface expression upon IFN-α treatment. As a control, we used HeLa cells that constitutively express highly glycosylated forms of BST-2/tetherin (Additional file 1: Figure S3B).

**Figure 2:**
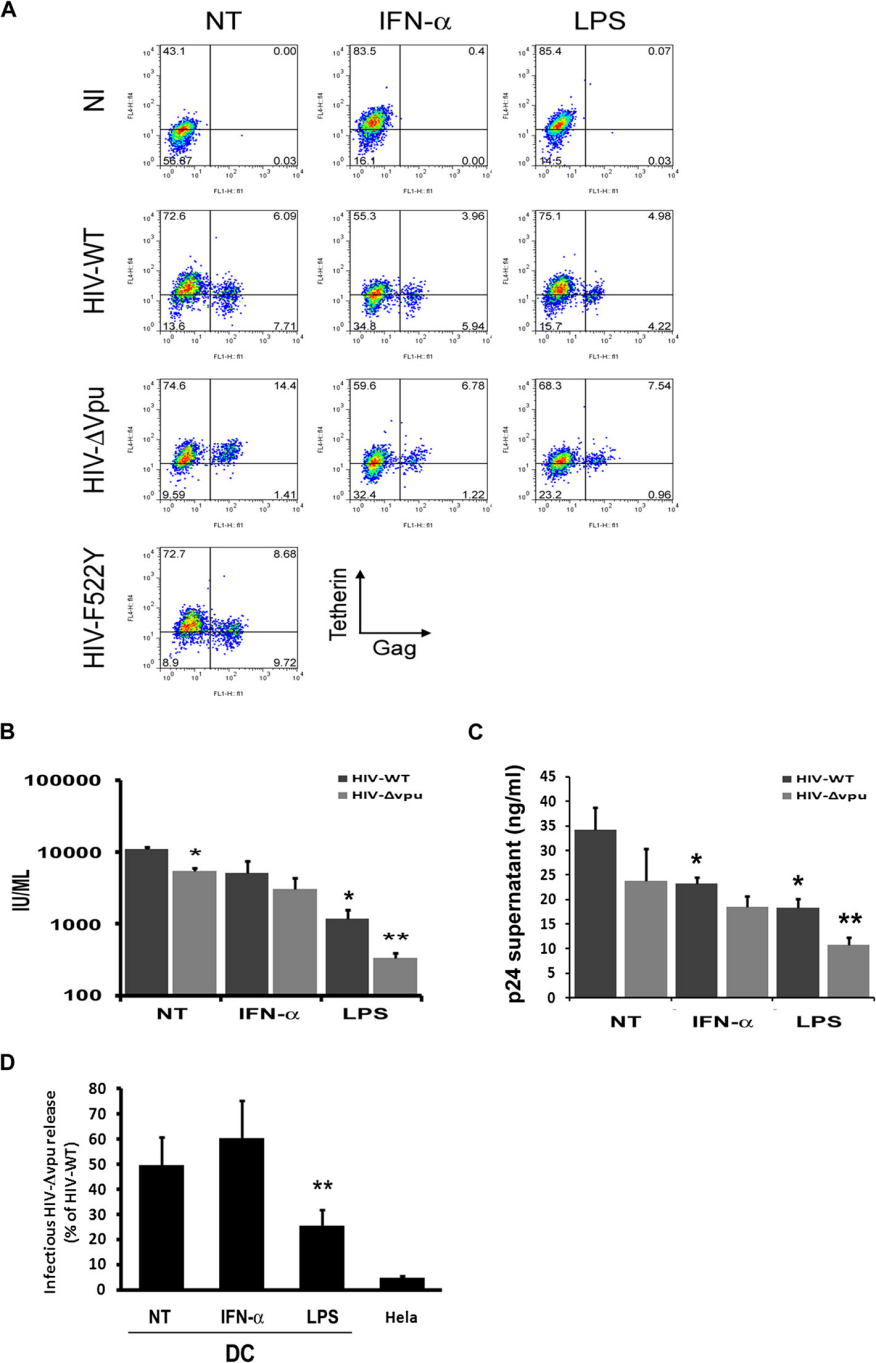
**LPS-induced BST-2/tetherin restriction activity in HIV-1 infected DC. (A)** 2 × 10^5^ DC, previously treated (or not) with IFN-α or LPS, were left uninfected (NI) or infected for 3 days with HIV-WT, HIV-ΔVpu or HIV-F522Y viruses in parallel with SIV3 (+Vpx) transduction. Infection rates in DC were scored by FACS analysis of BST-2/tetherin^+^/p24gag^+^ cells, three days post-infection. **(B)** TZM-*bl* cells were incubated for 48 hours with 1/10 volume of supernatant from HIV-WT or HIV-ΔVpu infected DC, previously untreated (NT) or treated with IFN-α or LPS, and harvested three days post-infection. Infectious units (IU/ml) were quantified as above in NT DC and treated DC (from 5 and 3 experiments respectively). **(C)** Supernatants from HIV-WT or HIV-ΔVpu infected DC, previously untreated (NT) or treated with IFN-α or LPS, were harvested three days post-infection and HIV-p24 levels were quantified by ELISA. **(D)** Graph represents the percentage of HIV-ΔVpu / HIV-WT infectivity ratios from treated DC as indicated and compared to Hela cells.

We then compared HIV-WT and HIV-ΔVpu infection rates in DC pre-treated with IFN-α or LPS. After 3 days of infection with both viral strains, we observed a similar ~2 fold reduction of intracellular HIV-Gag staining in IFN-α- and LPS- treated DC compared to non-treated cells (Figure 2A). Pre-treatment with IFN-α or LPS decreased rates of infection in DC [11] but did not alter Vpu-mediated BST-2/tetherin down-regulation which was comparable to control infected cells (Figure 2A). DC were challenged with a HIV-1 fusion defective mutant (HIV-F522Y), which prevents transmission of HIV to target cells even with a similar viral input and therefore serves as an important control for further DC-mediated HIV-1 transfer experiments (Figure 2A). Surprisingly, IFN-α pre-treatment only modestly affected HIV-ΔVpu viral release and infectivity levels compared to HIV-WT (Figure 2B and 2C). In sharp contrast, LPS pre-treatment was shown to impose a 10-fold decrease of HIV-WT infectivity yields (Figure 2B) and a 2-fold decrease of viral release (Figure 2C). Strikingly, viral release and infectivity upon HIV-ΔVpu challenge were decreased by 2 fold and 3.5 fold, respectively, after LPS pre-treatment when compared to HIV-WT (Figure 2B, 2C and 2D). These data demonstrate that LPS-driven DC maturation can induce a significantly more potent restriction to HIV-ΔVpu infection than IFN-α treatment.

**Figure 3:**
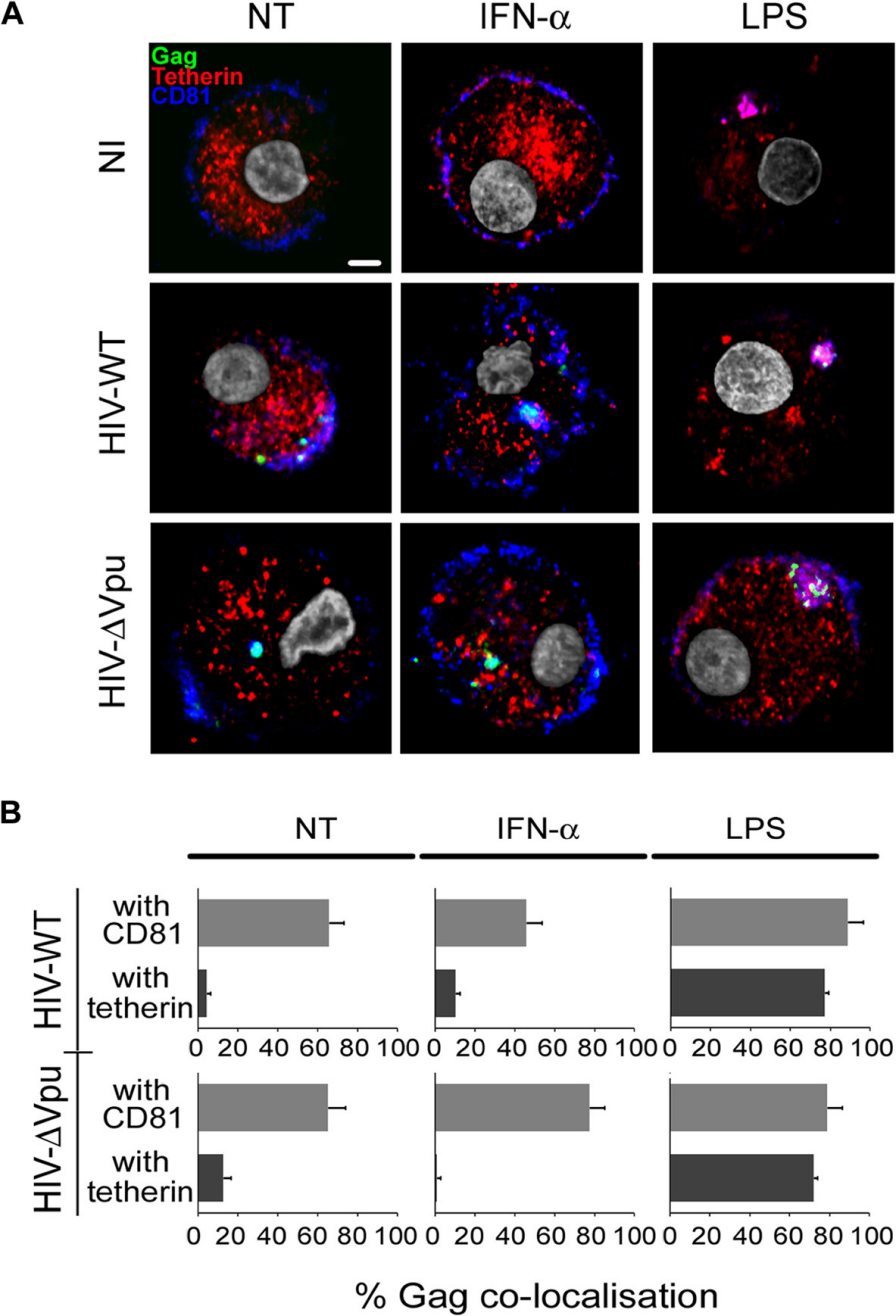
**LPS-induced relocalization of BST-2/tetherin in HIV-containing tetraspanin-enriched compartment. (A)** Confocal immunofluorescence analysis of HIV-Gag (green), BST-2/tetherin (red) and CD81 (blue) of DC pre-treated (or not) with IFN-α or LPS and left uninfected or challenged with HIV-WT or HIV-ΔVpu for 3 days. Data are representative of four independent experiments. Scale bars correspond to 5 μm. **(B)** Graphs represent the quantification of BST-2/tetherin and CD81 co-localisation with HIV-Gag from at least 24 cells per condition from 3 independent experiments.

To investigate the differential restriction of HIV-ΔVpu infection in DC after IFN-α or LPS, we analyzed BST-2/tetherin localization in infected DC. We observed that, while BST-2/tetherin was excluded from CD81-enriched domains in IFN-α pre-treated DC as seen in non-treated cells, LPS leads to a dramatic relocalization of the restriction factor to an intracellular CD81-enriched compartment (Figure 3A and 3B). After HIV challenge, BST-2/tetherin was also relocalized to the HIV-containing tetraspanin-enriched microdomain (TEM) in LPS-treated DC, while it remained excluded from this compartment in non-treated and IFN-α pre-treated infected DC (Figure 3A and 3B). These results highlighted a potential role of BST-2/tetherin on HIV-1 replication in LPS-mediated mature DC and prompted us to analyze if this could have any consequences on mature DC-mediated transfer of *cis* infection to CD4^+^ T cells compared to non-treated and IFN-α treated DC.

### BST-2/tetherin localization at the virological synapse and impact on DC-mediated HIV-1 transfer to CD4^+^ T Cells

**Figure 4:**
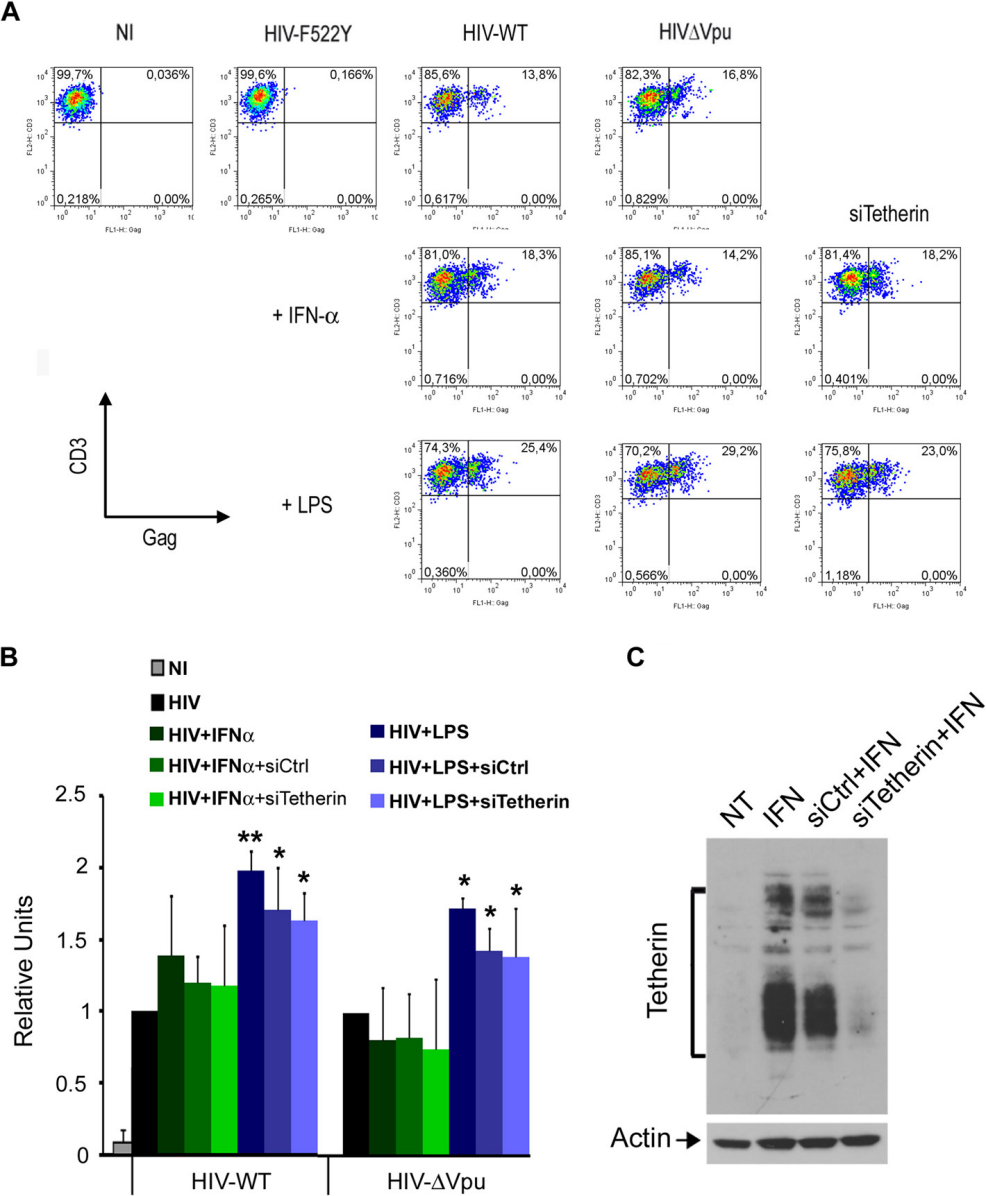
**BST-2/tetherin impact on DC-mediated HIV-1 transfer *in trans. *(A)**. 1 × 10^5^ DC, previously transfected (or not) with siTetherin or si Control (siCtr) for 48 hours, were left untreated (NT) or treated with IFN-α or LPS and challenged for six hours with 200 ng P24gag of HIV-ΔVpu or HIVF522Y viruses. DC, extensively washed, were then co-cultured with 1 × 10^5^ autologous CD4^+^ T cells pre-treated 30 minutes before co-culture with Indinavir. HIV-1 transfer on autologous CD4^+^ T cells was scored by FACS analysis of CD3^+^p24gag^+^FITC cells, four days post-transfer. Data shown are representative of three independent experiments. **(B)**. Fold enhancement of HIVWT and HIV-ΔVpu transfer in CD3^+^/CD4^+^ T cells. A value of 1 was assigned to the percentage of transfer in the non-treated (HIV) conditions. Error bars indicate standard error mean (SEM). Data are representative of four different experiments. P-values of paired bilateral Student Test were not significant (p>0.18). **(C)**. DC were transfected with siTetherin or siControl (siCtr) and left untreated (NT) or treated with IFN-α for 24 hours. Tetherin expression was analyzed by western blot. Data are representative of four experiments.

As DC play a critical role in virus dissemination by capturing HIV-1 and transferring the virus to T cells in a biphasic mode ([20,50] and reviewed in [20]), we first studied the effect of BST-2/tetherin in the initial transfer phase, or *trans* infectious mode. DC were left untreated or treated with IFN-α for 24h and challenged with HIV-1 for 6 hours. Upon DC-mediated HIV-1 capture, cells were co-incubated for 3–4 days with autologous CD4^+^ T cells pre-treated with indinavir to stop replication. We observed that IFN-α and LPS treatments did not impact significantly on DC-mediated transfer of captured HIV-WT or HIV-ΔVpu (Figure 4A and 4B). These results were also confirmed with Jurkat T cells used as targets (Additional file 1: Figure S4). Therefore, we aimed to look at BST-2/tetherin impact on DC-mediated HIV-1 transfer *in trans* by silencing the cellular restriction factor expression with specific targeted siRNA (siTetherin) compared to scrambled siRNA (siCtrl)-transfected DC (Figure 4C). As shown, HIV-1 transfer *in trans* from siTetherin-DC was similar to viral transfer from siCtrl-DC upon HIV-WT or HIV-ΔVpu capture (Figure 4A and 4B). As a control, we challenged DC with HIV-F522Y (introduced in Figure 2A) to rule out any HIV-1 transfer potentially derived from the initial viral input (Figure 4A).

**Figure 5:**
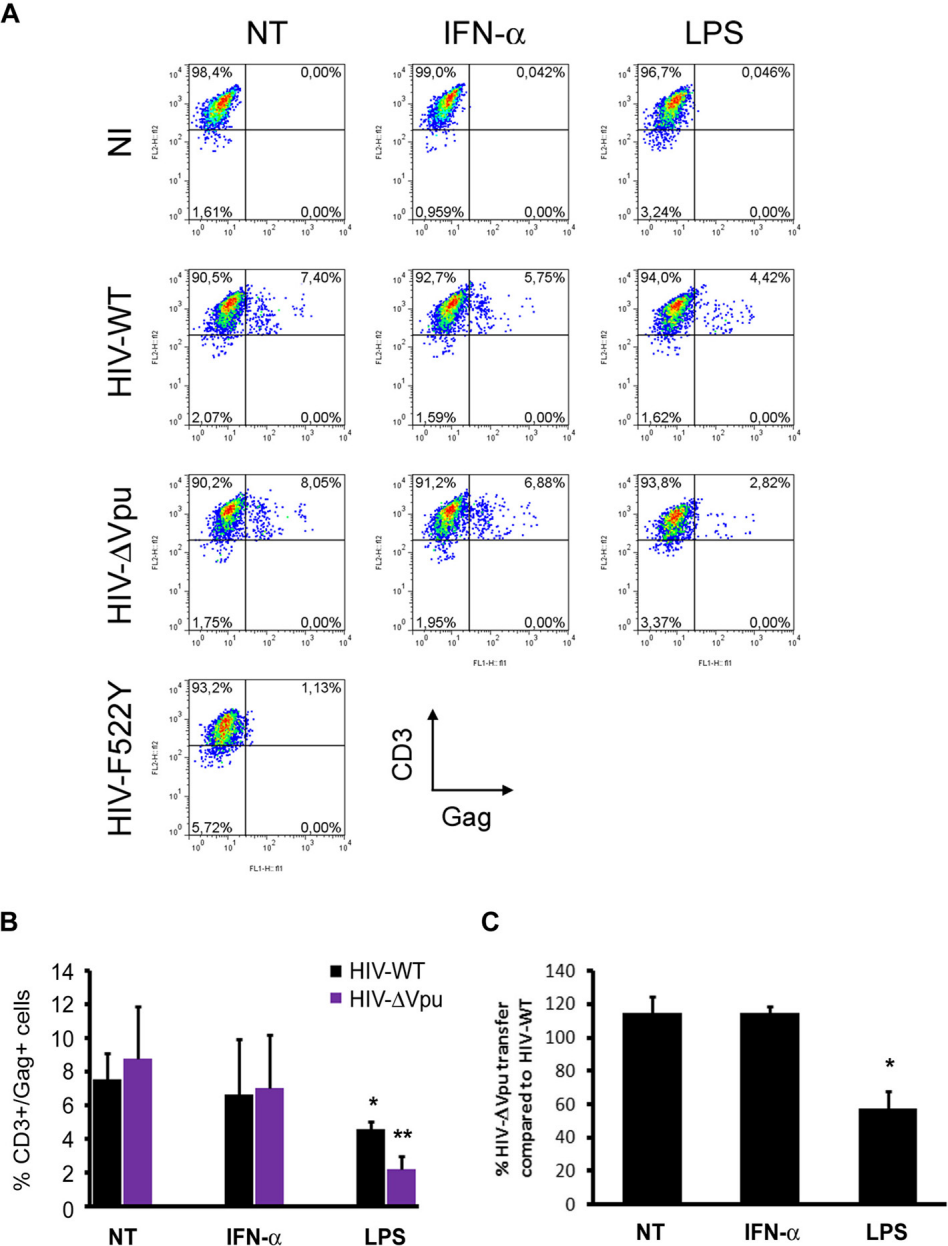
**BST-2/tetherin-mediated restriction on *cis*-infection from matured infected DC. (A)** 1 × 10^5^ DC, previously treated (or not) with IFN-α or LPS, were left uninfected (NI) or infected for 3 days with HIV-WT, HIV-ΔVpu or HIVF522Y viruses in parallel with Vpx-VLPs transduction. Cells were then co-cultured with 1 × 10^5^ autologous CD4^+^T lymphocytes. HIV-1 transfer on T lymphocytes was scored by FACS analysis of CD3^+^p24gag^+^FITC cells, three days post-transfer. **(B)** Quantification of viral transfer from treated DC as indicated was obtained from 5 different experiments for NT and IFN-α conditions and 3 independent experiments for LPS condition. **(C)** Percentage of DC-mediated HIV-ΔVpu transfer for each condition was then measured and depicted in the lower graph.

The potent restrictive activity of BST-2/tetherin against HIV-1 was shown to take place during late events of viral replication when budding occurs at the plasma membrane [24,25]. However, the potential impact of BST-2/tetherin on cell-to-cell viral spread appears controversial in recent reports analyzing HIV-1 transfer from T cell-to-T cell [43,51,52]. It was thus important to establish if BST-2/tetherin had a role in viral transfer to CD4^+^ T cells after HIV-1 replication in DC. Untreated, IFN-α and LPS-treated DC were infected for three days with HIV-WT or HIV-ΔVpu and then co-cultured with Indinavir-treated autologous CD4^+^ T cells for 3 additional days. FACS analysis of intracellular HIV-Gag in CD3^+^ cells revealed no major differences in the transfer rates of either virus whether the infection came from NT DC or IFN-α pre-treated DC (Figure 5A and 5B) despite comparable infection input (data not shown). Of note, a partial effect of IFN-α on HIV-1 transfer upon replication was also observed in a T cell-to-T cell model [53]. In each experiment, the fusion mutant viral strain (VSV-G pseudotyped-HIV-F522Y) that is able to complete only the first replication cycle, was used as a control to exclude any potential T cell infection coming from the initial viral input (Figure 5A). While mature DC were shown to efficiently capture and transfer HIV-1 in *trans* to target cells, HIV-1 replication is known to be potently altered in these cells thus limiting *in-cis* infection of CD4^+^ target T cells [18,50]. Indeed, we observed that LPS matured DC-mediated HIV-1 transfer in *cis* to CD4^+^ T cells was significantly reduced but with an even more pronounced deficit when mature DC were infected with HIV-ΔVpu (Figure 5C).

**Figure 6:**
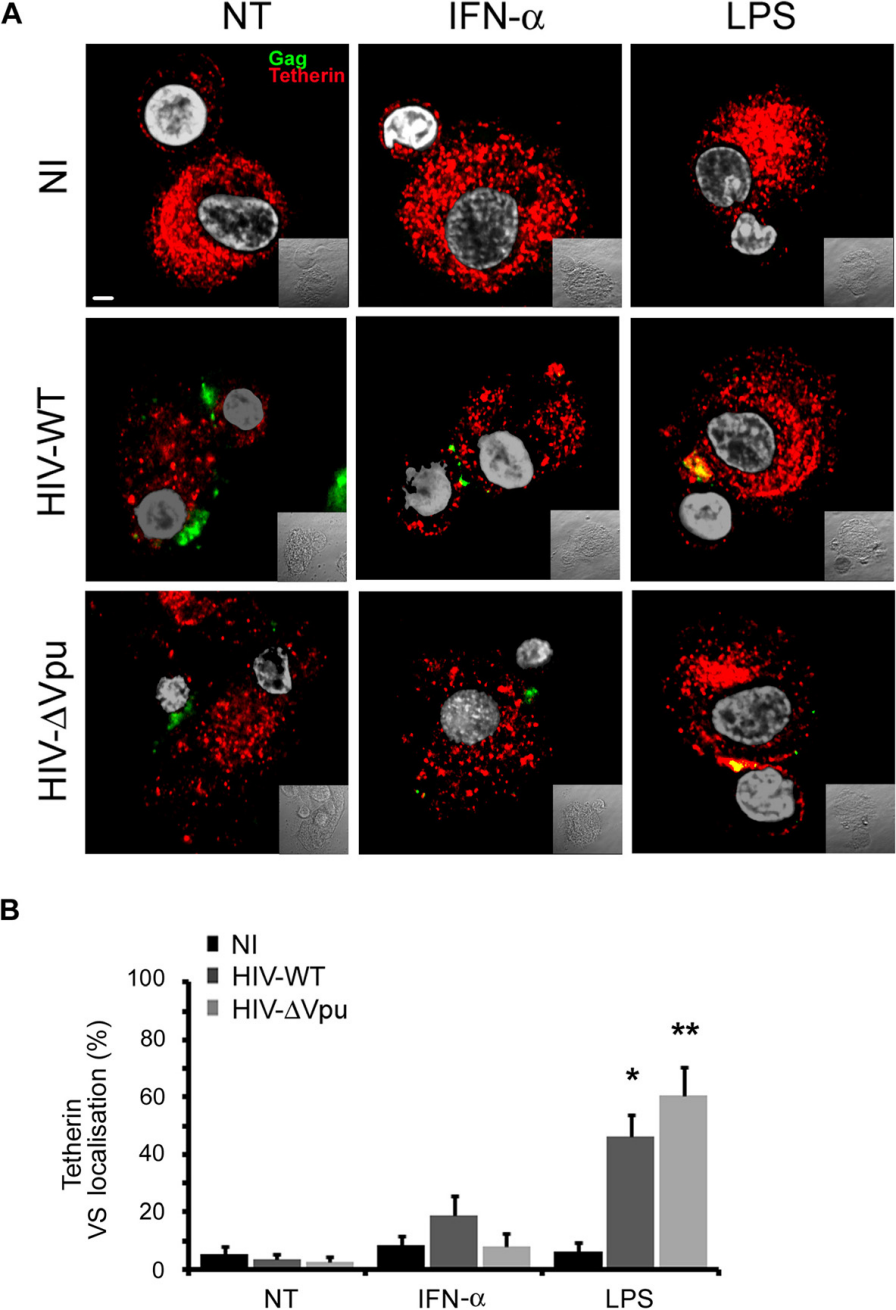
**BST-2/tetherin polarization and enrichment at the Virological Synapse. (A)** Confocal immunofluorescence analysis of HIV-Gag (green) and BST-2/tetherin (red) upon DC/T cell contacts from experiments above. **(B)** Virological Synapse (VS) signal intensity / total signal was then quantified from 3 different experiments (n= 15 for each condition) for each marker.

In the reported T cell-to-T cell transfer assays of HIV-1, BST-2/tetherin was shown to localize to, or even be enriched within, the virological synapse (VS) in order to inhibit or inversely potentiate Vpu-defective virus transfer respectively [43,51]. In order to explain the effect of BST-2/tetherin activity on viral transfer *in cis*, we then analyzed its localization at the interface of infected DC in contact with autologous CD4^+^ T cells. When polarized HIV-Gag was observed between T cells and NT or IFN-α treated DC, BST-2/tetherin was in fact drastically excluded from cell-to-cell contacts (Figure 6A) in sharp contrast with the striking enrichment of CD81 at the VS (Additional file 1: Figure S5A). A small amount (5-18%) of total membrane BST-2/tetherin was found to localize at the VS established between HIV-WT or HIV-ΔVpu infected NT or IFN-α treated DC (Figure 6B) whereas around 60% of membrane CD81 tetraspanin was found to be polarized at the DC-T VS (Additional file 1: Figure S5B). Strikingly, we found about 50-60% of the membrane BST-2/tetherin to be relocalized at the VS formed between infected LPS matured DC and CD4^+^ T cells, in parallel with an enhanced CD81 enrichment (Figure 6B and Additional file 1: Figure S5B).

## Conclusions

BST-2/tetherin was demonstrated to be a potent restriction factor for enveloped virus release [24-27,29,30]. However, its role in cell-to-cell viral transfer is still unclear. *In vivo* viral restriction activity of BST-2/tetherin was demonstrated in a report showing that tetherin-deficient mice could not inhibit Moloney murine leukemia virus (Mo-MLV) replication and disease progression [54]. While this restriction factor is constitutively expressed at low levels at the surface of some hematopoietic cells, its antiviral action is only unveiled after IFN-α induction and was, therefore, proposed to be critical for the antiretroviral activity of IFN-α *in vivo*[54]. This agrees with a recent report showing that induction of BST-2/tetherin expression after pegylated IFN-α/ribavirin treatment strongly correlated with reduction in HIV-1 load and with the antiretroviral capacity of IFN-α *in vivo*[55]. As an inflammatory environment is a pre-requisite to increase both BST-2/tetherin expression and restriction activity in specific cell types, we aimed to investigate the potential restriction activity of BST-2/tetherin in different DC subsets while modulating their maturational state with IFN-α or LPS. One of the limiting steps to studying HIV-1 infection in DC from myeloid origin is the fact that these cells are quite refractory to HIV-1 infection, more particularly when cells are matured, due to potent post-entry restriction blocks [6,7,10]. Taking advantage of the recent characterization of SAMHD1-mediated restriction in myeloid DC [6,7], we used Vpx-expressing lentiviral vectors in order to remove this restriction block and to allow higher levels of HIV-1 infection to be reached in DC from myeloid origin. This provided a more accurate way to measure the effects of BST-2/tetherin in this cell type. This setting did not compromise either DC maturation or the response to IFN-α or LPS treatment but allowed higher levels of HIV-1 infection, as previously shown [56] and as evidenced by BST-2/tetherin and RIG-I up-regulation. Interestingly, BST-2/tetherin up-regulation was recently proposed to occur in most immune cells from patients in the acute phase of HIV-1 infection [57] as well as in simian CD4^+^ T cells upon SIV infection [58]. In fact, and in agreement with a recent report from Coleman *et al*. [42], we found that, although HIV-1 infection led to BST-2/tetherin up-regulation, viral spread from DC was not efficiently restricted in the presence of BST-2/tetherin, even after removal of the SAMHD1-mediated post-entry restriction block with Vpx. Our evidence also showed that while constitutive BST-2/tetherin expression could be detected at the surface of both monocyte-derived DC and myDC, its expression was greatly up-regulated after IFN-α or LPS treatment of both DC subsets. When primary DC subsets were challenged with HIV-WT and HIV-ΔVpu, we observed that BST-2/tetherin surface expression could also be decreased in a Vpu-dependent manner as previously reported for other cells [24,25,28]. Surprisingly, however, Vpu-mediated down-regulation of BST-2/tetherin from the cell surface did not correlate with significant enhancement of viral release suggesting that the lack of tetherin-mediated HIV-1 restriction might be Vpu-independent. Hence, BST-2/tetherin down-regulation at the cell surface of both DC subsets was not always followed by its degradation, as previously observed in other cells [37,38,48]. Overall, these results demonstrated that the absence of Vpu did not result in significant alteration of HIV-1 replication in these cells and thus precluded BST-2/tetherin-mediated restriction activity against HIV-1 in primary DC.

Interestingly, some recent reports have shown that BST-2/tetherin expression is up-regulated after stimulation with TLR agonists [42,57,59,60], which reinforces the idea that this cellular restriction factor could be a critical component of the intrinsic innate immune response. We have demonstrated here that, while IFN-α treatment was unable to efficiently restrict DC-to-T cell HIV-1 transfer, LPS-mediated maturation of DC induced a much more potent blockade of viral infection and transfer *in-cis* toward autologous CD4^+^ T cells. Strikingly, when LPS-matured DC were challenged with HIV-ΔVpu, viral replication and transfer were decreased by more than 4 fold and 2 fold (respectively) compared to HIV-WT.

As tetherin surface levels were modulated in IFN-α or LPS-treated cells and given that previously described results highlighted a more potent restriction of HIV-ΔVpu in LPS treated DC compared to IFN-α treated DC, we hypothesized that a potential mechanism of BST-2/tetherin restriction in DC could rely on its cellular localization rather than overall expression. Additionally, treatment with IFN-α or a TLR agonist was shown to differentially modulate DC maturation and immune function. In comparison to LPS stimulation, IFN-α was proposed to only partially affect DC maturation [61,62] while also inhibiting the production of IL-12 and thus potentially skewing the T helper response [63,64]. However, IFN-α is considered as a major regulator of the antiviral response and has been shown to be a potent inducer of CD8^+^T cell cross-priming and therefore critically required to induce DC full maturation upon engagement of a TLR [65-67]. One of the marked differences between both of these biological compounds in the process of DC maturation is that, while LPS-driven maturation is followed by a decreased rate of endocytosis, antigen-processing and a stabilization of MHC-II/peptide complexes, IFN-α does not alter MHC-II synthesis, antigen processing or endocytosis rates [68,69]. Thus, vesicular trafficking pathways could behave differently depending on the applied stimulus to mature DC. In fact, we observed that BST-2/tetherin was relocalized to tetraspanin-enriched compartments only after LPS stimulation. This localization was similar to the HIV-containing tetraspanin-rich compartment previously described [46,70,71]. When LPS-matured DC were infected with HIV-1, we observed that BST-2/tetherin was co-localized with tetraspanin CD81. In contrast, BST-2/tetherin did not accumulate significantly with CD81 in IFN-α matured DC.

The intracellular relocalization of BST-2/tetherin observed in DC after LPS treatment did not influence DC-mediated viral transfer in *trans* toward CD4^+^ T cells. This was demonstrated by the comparable data obtained with DC-mediated HIV-WT and HIV-ΔVpu transfer of infection to CD4^+^ T cells. This was also confirmed when BST-2/tetherin expression was knock-downed in DC loaded with HIV-1. Of note, LPS pre-treatment of DC even increased DC-mediated HIV-1 transfer in *trans*, independently of BST-2/tetherin expression as reported by Coleman et al. [42]. While in agreement with the previously described model of increased HIV-1 transmission by mature DC through *trans*-infection [19,42,72-74], our data exclude a BST-2/tetherin effects during early DC-mediated HIV-1 capture and transfer to CD4^+^ T cells.

In contrast, our data with LPS pre-treated DC suggested that tetherin potently restricts DC-mediated *cis*-infection of CD4^+^ T cells. This restriction correlated with the polarization of BST-2/tetherin into the HIV-1-containing tetraspanin-enriched compartments which is part of the trafficking pathway required for DC-T cell transfer during HIV-1 infection [46]. A plausible hypothesis would be that this virus-containing compartment, previously thought to be an escape route for HIV-1, could in fact become an innate antiviral cell host defense mechanism when fueled with BST-2/tetherin molecules after LPS-mediated maturation of DC.

In agreement with its restrictive action in LPS-matured DC-mediated viral transfer in *cis*, we found BST-2/tetherin relocalized to the virological synapse along with HIV-1 and TEM markers. Hence, it appeared that BST-2/tetherin localization at the virological synapse was further enhanced with HIV-Δvpu infected LPS-matured DC in contact with CD4^+^ T cells, which correlated with the increased accumulation of BST-2/tetherin observed in HIV-1 containing TEM within LPS-matured DC infected with HIV-Δvpu. These results also suggest that, while the lack of BST-2/tetherin-mediated HIV-1 restriction observed in DC-mediated trans-infection appeared to be Vpu-independent, Vpu might indirectly contribute to overcome BST-2/tetherin antiviral activity on DC-mediated HIV *cis*-infection toward CD4^+^ T cells. Indeed, in the absence of Vpu, more BST-2/tetherin molecules from the cell surface are available when polarised into the HIV-containing compartment upon LPS treatment, reinforcing the retention of *de novo* budding virions and the restriction of *vpu*-deficient HIV-1 transmission compared to wild-type viruses. However, as evidenced by our data, the presence of Vpu in infected DC does not fully overcome BST-2/tetherin antiviral activity. Interestingly, a recent publication [75] reported that localisation of BST-2/tetherin to the virus-containing compartment (VCC) of infected macrophages correlated with a decreased HIV release and cell-to-cell transmission. Importantly, this report also suggested that BST-2/tetherin was essential for VCC formation. If BST-2/tetherin molecules at the cell surface are a major source for the virus-containing compartment, this could explain the apparent increased size of the HIV-containing compartment observed in HIV-ΔVpu infected DC.

As shown here, and as previously reported [42,76], BST-2/tetherin is expressed at low levels on DC from myeloid origin but its expression is increased after HIV-1 infection [57]. Indeed, conditions where cytokines and/or pathogen associated molecular patterns (PAMPs) could up-regulate BST-2/tetherin expression would fit with the concept of the dysregulated cytokine storm observed during HIV-1 acute infection (for review see [77]). Furthermore, this might even be exacerbated during co-infection events engaging specific TLR pathways. Surprisingly, however, we found that IFN-α and TLR agonist treatments were both differentially regulating BST-2/tetherin intracellular trafficking.

The lack of BST-2/tetherin restriction in IFN-α treated DC could be due to the complex trafficking pathway used by HIV-1 in DC for viral assembly and release. Indeed, in contrast to T cells and some epithelial cell line models in which viral release occurs mainly at the plasma membrane, HIV-1 budding and release from DC and macrophages was shown to take place in complex compartments connected, at least in part, to the cell surface and enriched in tetraspanins [46,70,78-80]. Therefore, in conclusion, we demonstrated that the innate antiviral BST-2/tetherin activity could be modulated depending on the context of DC maturational state and inflammatory environment.

## Methods

### Ethics statement

All patient samples and cell protocols were approved by the ethical committees of the Universities of Geneva and Cardiff. Written informed consent was provided by study participants and validated by the institutional review board.

### Cells and reagents

Primary cells were purified from peripheral blood mononuclear cells (PBMC) of healthy donors by magnetic bead-mediated selection, according to the manufacturer’s instructions (Miltenyi Biotech). Monocytes were purified with CD14 MicroBeads and cultured for 5 days in IMDM supplemented with 10% FCS, 100 U/ml penicillin, 100 μg/ml streptomycin, 500 U/ml GM-CSF and 500 U/ml IL4 (Strathman Biotec, Germany) to generate immature monocyte-derived DC (MDDC), as previously described [3]. MyDC were purified with a CD1c DC kit and cultured in IMDM, supplemented with 10% human serum and 250 U/ml GM-CSF. Resting CD4^+^T cells were isolated with a CD4^+^T cell isolation kit II and cultured in RPMI 1640 supplemented with 10% FCS, 100 U/ml penicillin, and 100 μg/ml streptomycin. They were activated with 5 μg/ml phytohemagglutinin (PHA) for 36 hours and then maintained by adding IL2 (20 U/ml Roche). 293T human embryonic kidney cells, TZM-bl cells and HeLa P4-R5 cells (NIH AIDS Research & Reference Reagent Program) were maintained in supplemented DMEM. HIV-p24 was quantified in supernatants from infected DC using a p24 ELISA kit (SAIC-Frederick, Maryland, USA). When stated, MDDC were pre-treated with 1000 U/ml interferon alpha (IFN-α, Sigma) for 24 hours or 100 ng/ml LPS.

### Viral stock production

Viral stocks were generated by transient transfection of HEK293T cells with plasmids encoding either full-length X4-tropic HIV-WT (pR9 and pNL4.3) or X4-tropic *vpu*-deficient virus (HIV-ΔVpu) (pR9*-Δvpu* and pNL4.3*-Δvpu*) (both plasmid provided by Trono D, EPFL, Lausanne). VSV-G pseudotyped HIVF522Y was produced by co-transfection of the MD.G plasmid [81] containing the vesicular stomatis virus g-glycoprotein envelope (VSVG) and a plasmid encoding full length HIV-X4 genome with non fusogenic gp120/gp41 complex [82]. Infectious titers of viral stocks were evaluated by limiting dilution on HeLa P-4.2 cells containing a β*-gal* gene under the control of HIV-1 long terminal repeat (LTR). Physical particles were evaluated by quantification of HIV-1 p24gag by ELISA kit (Beckman Coulter, Paris, France). VSVG-Pseudotyped SIV3 lentivector encoding the Vpx gene [12,83] was obtained by co-transfection of SIV3^+^ packaging construct and pMD.G. The physical titer of SIV3 was quantified by exogenous reverse transcriptase (exo-RT) assay against standards of known activity.

### Antibodies

Rabbit anti-BST2 made by K. Strebel [49] was obtained through the AIDS Research and Reference Reagent Program (Division of AIDS, NIAID, NIH, Bethesda, USA) and was used for immunoblotting and immunofluorescence assays. Goat anti-actin, HRP conjugated horse anti-rabbit and anti-goat (Bio-Rad) were used for immunoblotting. Mouse anti-tetherin used for FACS analysis was from Chugai. Human anti-CD3 and anti-CD81 were from BD Pharmingen and anti-P24gag FITC (Kc57 FITC) was from Coulter.

### Infection and transfer

MyDC or MDDC (1 × 10^5^ cells) were added to 96-well plates in 100 μl IMDM for challenge with either HIV-WT (5 ng p24Gag) or HIV-ΔVpu (50 ng p24Gag) together with 10000 cpm of Vpx-expressing lentivector. MyDC and MDDC were washed after 24 hours of infection and maintained at 37°C for another two days. HIV-1 transfer *in cis* was done by co-culturing infected MDDC for three days with 1 × 10^5^ CD4^+^ T cells pre-treated with Indinavir (2 μg/ml). DC-mediated HIV-1 transfer *in trans* was done as previously described [3].

### Flow cytometry

After isolation or differentiation, surface staining of primary cells were performed as previously described. Productive infection of a pool of myDC and MDDC was measured by flow cytometry analysis of HIV-Gag^+^ cells, 3 days post-infection, using an anti-tetherin antibody as a surface marker. Cells were then washed, fixed, permeabilized and stained with anti-p24gag-FITC and analyzed on a FACS calibur (Beckton Dickinson). Productive viral transfer in CD4^+^T cells was controlled by flow cytometry analysis of intracellular HIV-Gag^+^ cells, 3 days post-transfer, using an anti-CD3 antibody as a T cell surface marker.

### Confocal immunofluorescence

Cells were left to adhere on poly-L-lysine treated glass coverslips for 60 min at 37°C. Cells were then fixed, permeabilized and stained with the indicated antibodies followed by fluorescently labeled secondary antibodies (Jackson ImmunoResearch Laboratories, West Grove, PA). Staining of Gag, tetherin and CD81 were done using anti-Gagp24 KC57-FITC, anti-BST-2 pAb (from K. Strebel) and anti-CD81 mAb respectively. Confocal microscopy analysis was carried out on a Zeiss LSM510Meta using a 63× objective and green, red and far red fluorescence were acquired sequentially. Quantification of tetherin, CD81 and Gag colocalization events were performed using ImageJ Software (NIH) and analyzed with excel software (Microsoft).

### RNA interference in MDDC

MDDC (8 × 10^5^ cells) were plated per well (24-well plate) and transfected twice with 600 nM of a pooled siRNA targeting BST-2/tetherin (siBST2) (ON-TARGETplus SMARTpool) or with an siRNA control (siGenome Non-Targeting siRNA #5) (both from Dharmacon) using HiPerFect reagent (Qiagen), according to the manufacturer’s recommendations. A second round of transfection was performed 24 hours later. After 72 hours, expression knockdown was checked by Western blotting followed by densitometry analysis (Quantity One; Bio-Rad Laboratories), and the cells were subsequently used for infection assays.

### Statistical analysis

Significant differences between groups were calculated using the Student’s T test (*P*-values < 0.05 or <0.005 were considered significant and marked with (*) or (**) respectively).

## Misc

Fabien P Blanchet and Romaine Stalder contributed equally to this work.

## Competing interest

The authors declare that they have no competing interests.

## Authors’ contributions

FPB, RS, ML, BM and VP conceived the study and designed experiments; FPB, RS, MC, ML, LR and BM carried out experiments; FPB, RS and VP wrote the manuscript. All authors read and commented on the manuscript. This work was supported by Swiss National Science Foundation grants PP00A3-114755, The Human Science Frontier Program and Cardiff University to V.P. All authors read and approved the final manuscript.

## Supplementary Material

Additional file 1 **Figure S1.** Shows the same type of experiment as in Figure 1 but using primary myeloid DC (myDC) isolated from blood. **Figure S2.** Shows the infection level in DC at 2 and 5 dpi from cells challenged with HIV-GFP concomitantly with VLP-Vpx transduction. **Figure S3.** Shows the expression of BST-2/tetherin by FACS analysis (S2A) or immunoblotting (S2B) in lysates obtained from DC left untreated or treated with IFN-α or LPS. Comparison was made against tetherin level of expression in IFN-α-treated 293T cells or untreated Hela cells. **Figure S4.** Shows a representative FACS analysis similar to Figure 4 but with a CD4^+^ Jurkat T cell line as target cells. **Figure S5.** Shows confocal immunofluorescence data from Figure 6 but staining for HIV-Gag and CD81. (DOCX 13 kb) Click here for file
