# Supplementary material for: TLR-4 engagement of dendritic cells confers a BST-2/tetherin-mediated restriction of HIV-1 infection to CD4+ T cells across the virological synapse
Source: Retrovirology. 2013 Jan 11;10:6. doi: 10.1186/1742-4690-10-6 (PMC3561259; doi:10.1186/1742-4690-10-6)

**Supplemental Figure legends**

**Figure S1. Vpu-dependent BST-2/tetherin cell surface down-regulation but lack of BST-2/tetherin restriction activity in myDC**

**(A)**. 2 x10^5^ myDC (A, upper panel) and Vpx-transduced DC (A, lower panel) were infected or not (NI) with HIV-WT or HIV-ΔVpu viruses. Infection of DC was scored by FACS analysis of BST-2/tetherin^+^/p24gag^+^ cells, three days post-infection. Percentage of cell surface BST-2/tetherin^+^ in infected DC from 3 independent experiments was calculated (right graph panels). **(B)** Lysates and supernatants of uninfected (NI), HIV-WT or HIV-ΔVpu infected myDC were subjected to immunoblotting with anti-Gag and anti-BST-2. The presence of Vpu was assessed by immunoblotting with anti-Vpu and anti-actin served as loading control.


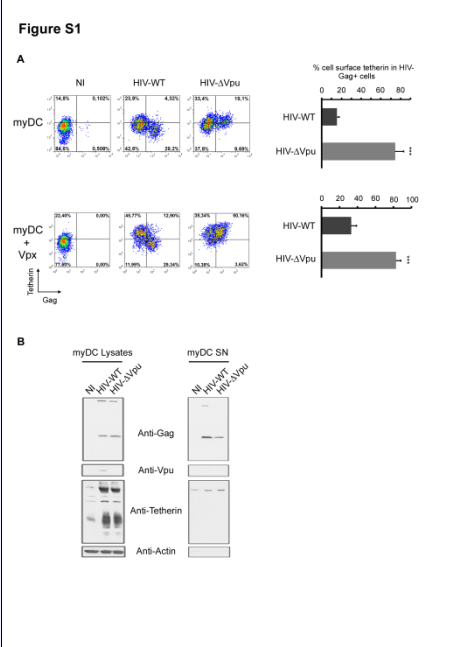


**Figure S2. Control of Vpx-expressing SIV-derived lentivector (SIV3) on HIV infection of DC**

2x10^5^ DC, previously transduced (or not) with 2 different doses of SIV3 (+Vpx), were challenged with pseudotyped HIV-GFP lentivector at a MOI of 0.5. GFP signal was scored by FACS two and five days post-infection.


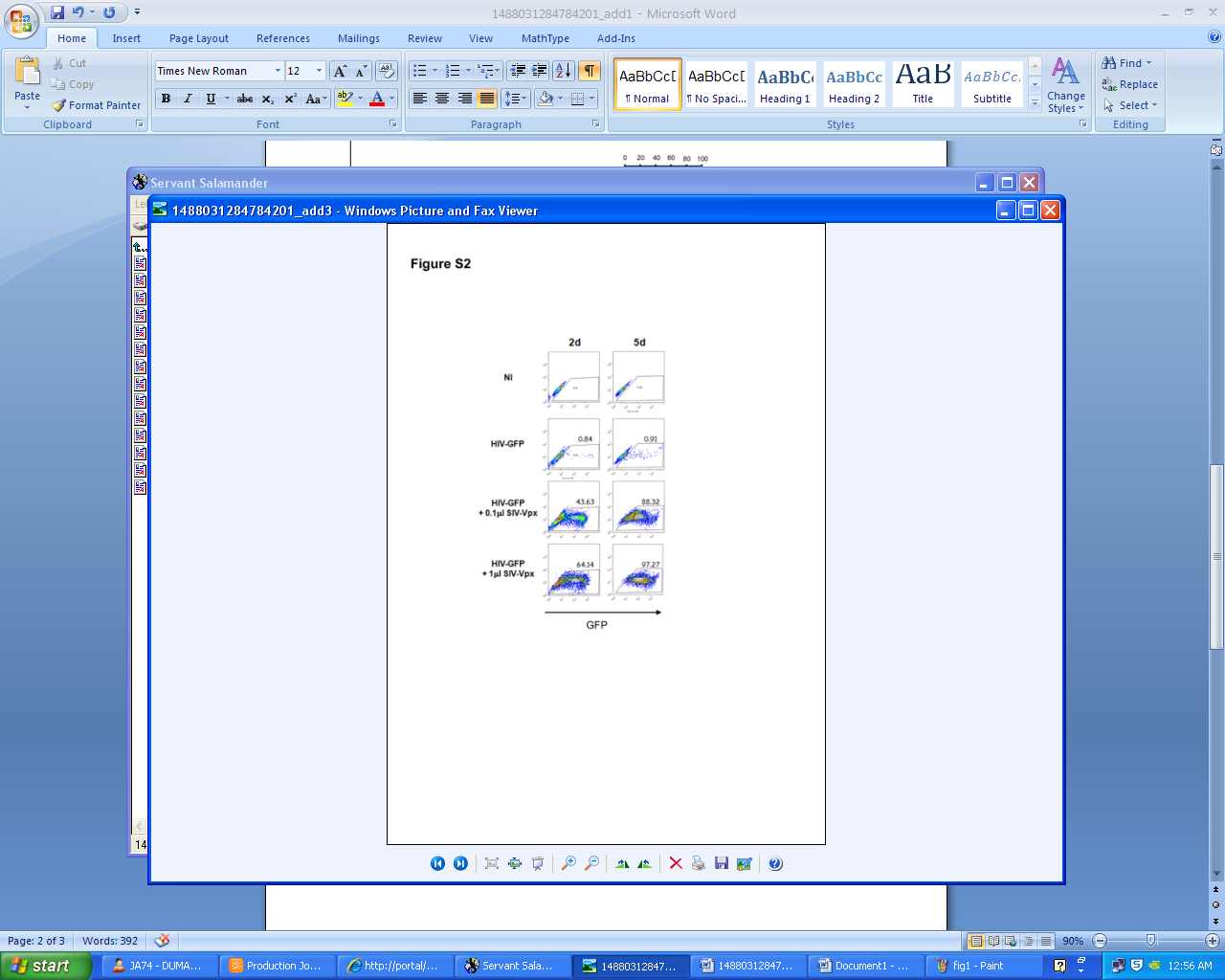


**Figure S3. BST-2/tetherin expression in DC**

**(A)** Cell surface (left panels) or intracellular (right panels) BST-2/tetherin expression was analysed on DC left untreated (NT in blue) or treated with IFN-α (in green) or LPS (in red) after 20h. Controls for antibody specificity and IFN-α activity were done using 293T cells. (B) Immunoblotting analysis of BST-2/tetherin expression in lysates of untreated, IFN-a-treated or LPS-treated DC was done using a rabbit anti-human BST-2/tetherin. Immunoblotting control for antibody specificity was done using 293T cells (untreated or treated for 20h with IFN-α) and Hela cells. Loading control was done using anti-actin.

**
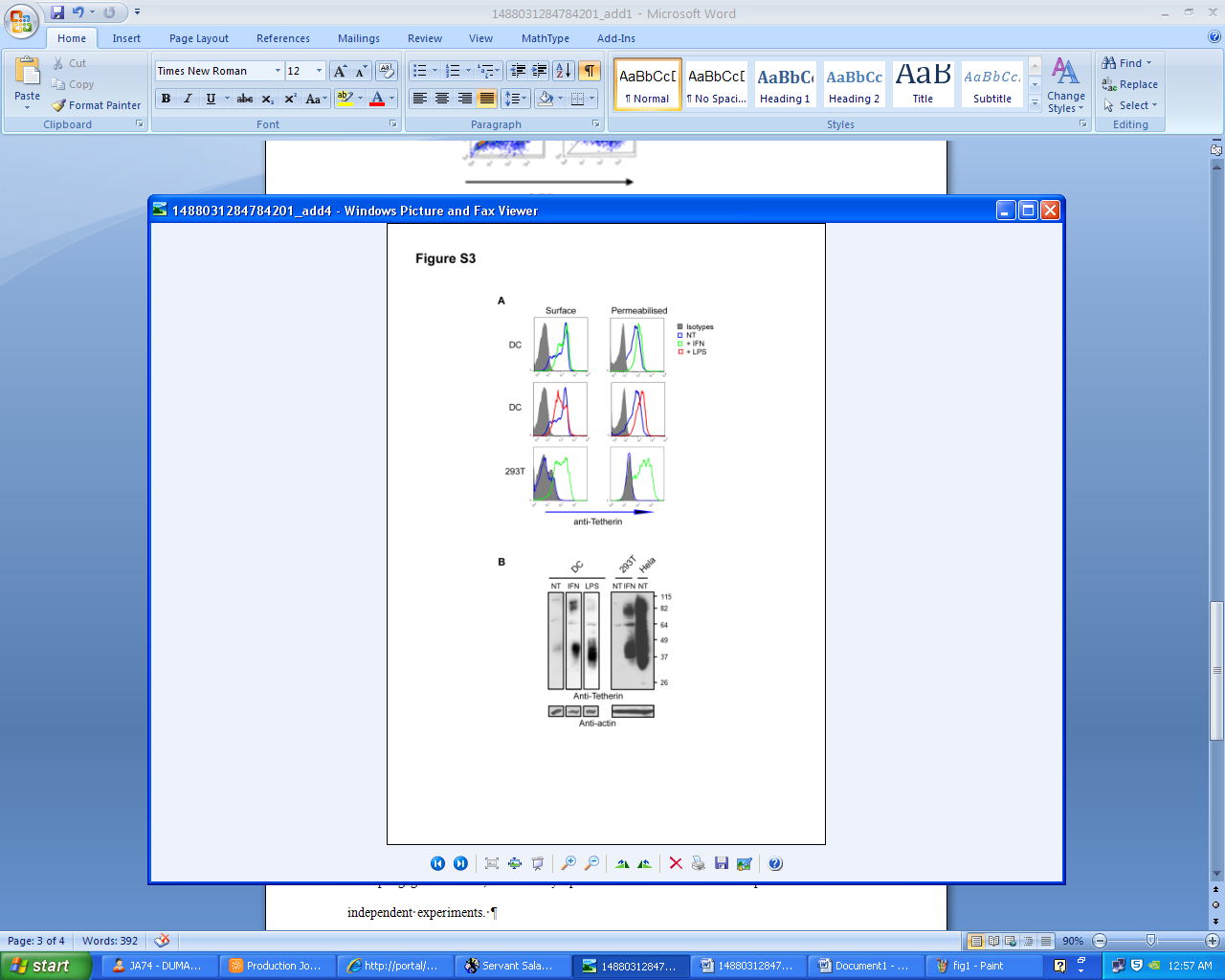
**

**Figure S4. BST-2/tetherin impact on DC-mediated HIV-1 transfer to Jurkat T cells *in trans***

**(A)**. 1x10^5^ DC were left untreated (NT) or treated with IFN-α or LPS and challenged for six hours with 200 ng P24gag of HIV−ΔVpu or HIVF522Y viruses. DC, extensively washed, were then co-cultured with 1x10^5^ CD4^+^ Jurkat T cells pre-treated 30 minutes before co-culture with Indinavir. HIV-1 transfer on T cells was scored by FACS analysis of CD3^+^p24gag^+^FITC cells, 3 or 4 days post-transfer. Data shown are representative of four independent experiments.

**
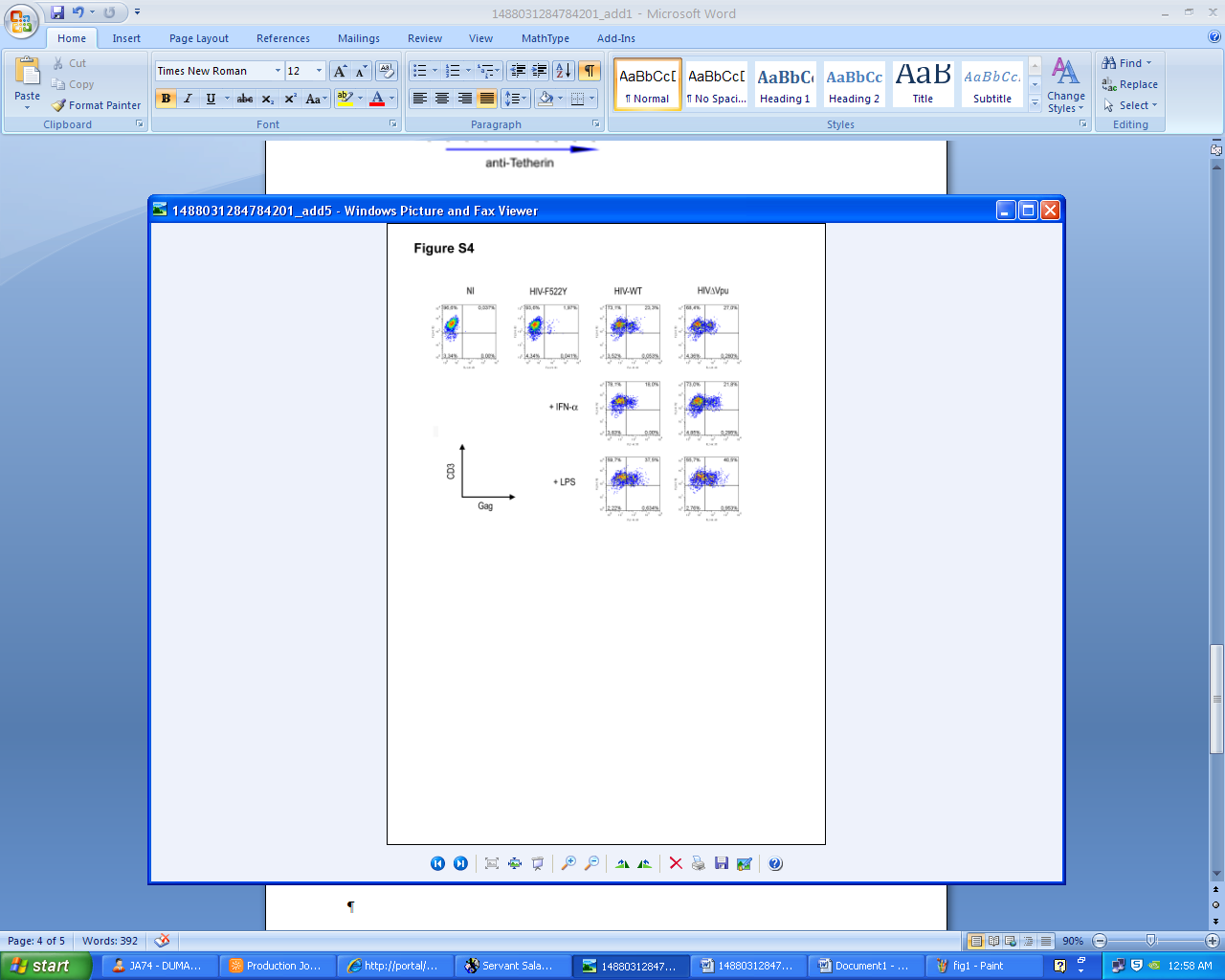
**

**Figure S5. CD81 polarization and enrichment at the Virological Synapse**

**(A)** Confocal immunofluorescence analysis of HIV-gag (green) and CD81 (blue) upon DC/T cell contacts from experiments represented in Figure 6. **(B)** Virological Synapse (VS) signal intensity / total signal was then quantified from 3 different experiments (n= 15 for each condition) for CD81.


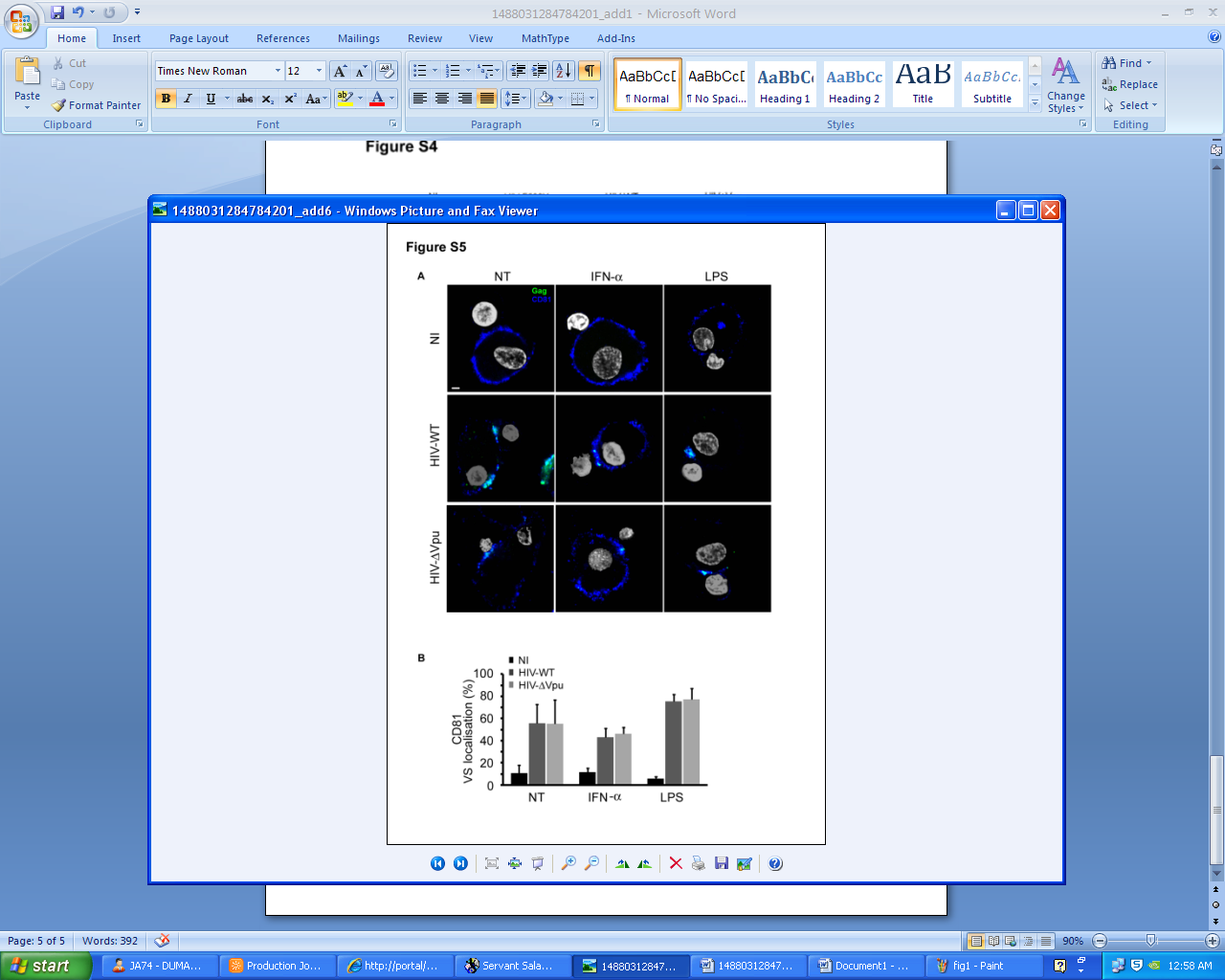

Supplement: Additional file 1 — Figure S1. Shows the same type of experiment as in Figure 1 but using primary myeloid DC (myDC) isolated from blood. Figure S2. Shows the infection level in DC at 2 and 5 dpi from cells challenged with HIV-GFP concomitantly with VLP-Vpx transduction. Figure S3. Shows the expression of BST-2/tetherin by FACS analysis (S2A) or immunoblotting (S2B) in lysates obtained from DC left untreated or treated with IFN-α or LPS. Comparison was made against tetherin level of expression in IFN-α-treated 293T cells or untreated Hela cells. Figure S4. Shows a representative FACS analysis similar to Figure 4 but with a CD4+ Jurkat T cell line as target cells. Figure S5. Shows confocal immunofluorescence data from Figure 6 but staining for HIV-Gag and CD81. (DOCX 13 kb) [file 1742-4690-10-6-S1.docx]
